# Primary Hepatic Histiocytic Sarcoma Mimicking Hepatocellular Carcinoma

**DOI:** 10.14309/crj.0000000000002229

**Published:** 2026-07-03

**Authors:** Evgenia Tziava, Aikaterini Leventi, Maria Arnaouti, Nektarios Ntalakos, Maria Igoumenidi, Dimitris P. Korkolis

**Affiliations:** 1 Department of Surgical Oncology, Agios Savvas Anticancer Hospital, Athens, Greece; 2 Department of Pathology, Agios Savvas Anticancer Hospital, Athens, Greece

**Keywords:** liver tumors, liver sarcoma, histiocytic sarcoma

## Abstract

Primary hepatic histiocytic sarcoma (HS) is an exceptionally rare malignancy with nonspecific clinical and morphological features that often mimic other high-grade liver tumors making their diagnosis challenging. We report a 74-year-old man presenting with abdominal and back pain, fatigue, and a solitary right-lobe hepatic mass. Imaging suggested hepatocellular carcinoma, and the patient underwent right posterior sectionectomy. Histopathology revealed a high-grade neoplasm of discohesive large cells with eosinophilic cytoplasm and extensive necrosis. Immunohistochemistry confirmed HS. Given the extreme rarity of primary hepatic HS, we provide a focused literature review highlighting diagnostic challenges, immunophenotypic features, and current insights into the molecular landscape of this aggressive neoplasm.

## INTRODUCTION

Histiocytic sarcoma (HS) is an exceptionally rare hematopoietic malignancy characterized by morphological and immunophenotypic features of mature tissue histiocytes. In the current World Health Organization (WHO) classification, HS is defined by these histiocytic traits, although it is not considered a true sarcoma.^1^ The overall incidence is estimated at approximately 0.17 cases per 1 million individuals per year^2^ underscoring its extreme rarity. Primary hepatic involvement is even less common, with only isolated case reports and small case series available, making liver presentation one of the rarest extra nodal manifestations of HS.^1^ Owing to the rarity of the diagnosis its clinical, radiologic, and histopathologic features remain poorly characterized. We present here a case of a 74-year-old man who was surgically treated for a large liver tumor mimicking a hepatocellular carcinoma (HCC) and proved to be a HS. This case contributes to the limited literature by highlighting the diagnostic challenges, the need for a broad differential in atypical hepatic lesions, and the importance of integrating imaging, pathology, and immunophenotyping to establish an accurate diagnosis.

## CASE REPORT

A 74-year-old man with a large right posterior hepatic mass was admitted for surgical evaluation. His history included myocardial infarction treated with coronary stents and chronic obstructive pulmonary disease. He reported dull abdominal and back pain with persistent fatigue for 6 months before presentation. Routine laboratory tests and tumor markers (carcinoembryonic antigen, carbohydrate antigen, carbohydrate antigen 19‑9, and alpha‑fetoprotein) were normal, and physical examination was unremarkable.

Ultrasonography revealed a round, homogeneous mass with hypoechoic margins. Magnetic resonance imaging (MRI) demonstrated an exophytic, lobulated lesion in segment VI measuring 5.3 × 5.0 × 4.9 cm, showing T2 hyperintensity, T1 hypointensity, microcystic degeneration, and restricted diffusion (Figure 1). Postcontrast imaging showed predominantly peripheral, irregular enhancement with a bilobed configuration and a feeding branch from the hepatic artery, along with mild adjacent parenchymal blurring. According to Liver Imaging Reporting and Data System (LI-RADS) v2018, the absence of nonrim arterial phase hyperenhancement and nonperipheral washout, combined with the presence of peripheral, irregular enhancement, classifies the lesion as LI-RADS category M (LR‑M). The overall appearance was therefore suspicious for malignancy, including but not limited to HCC.

**Figure 1.**
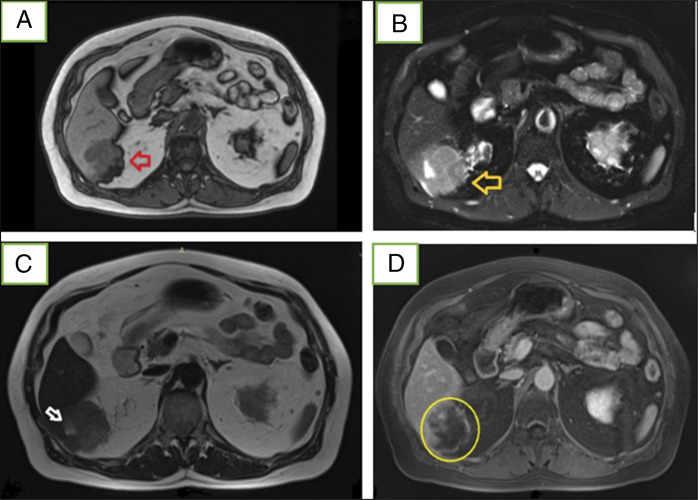
Magnetic resonance imaging of the liver demonstrating an exophytic lesion in the inner posterolateral peripheral region of segment VI. (A) T1-weighted sequence showing decreased signal intensity of the lesion (red arrow). (B) T2-weighted sequence demonstrating slightly increased signal intensity relative to the surrounding parenchyma (yellow arrow). (C) T2-weighted fat-suppressed sequence highlighting internal microcystic areas (white arrow). (D) Contrast-enhanced T2-weighted sequence after gadolinium administration, showing a lobulated contour and focal areas of restricted diffusion (yellow circle).

Given the lesion's surgical accessibility and the risk of tumor seeding, percutaneous biopsy was not pursued. The multidisciplinary tumor board recommended exploratory laparotomy and resection. The patient underwent right posterior sectionectomy (segments VI and VII) with cholecystectomy, achieving R0 margins. The specimen contained a multilobulated, firm, whitish-gray mass measuring 7.5 × 5.0 cm with focal necrosis and hemorrhage, extending into surrounding adipose tissue (Figure 2).

**Figure 2.**
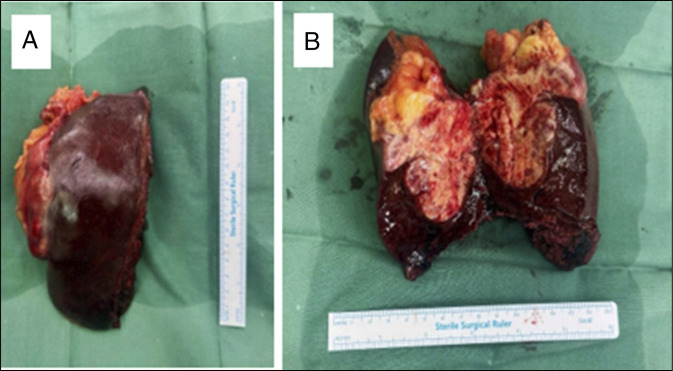
Gross pathology of the resected hepatic specimen. (A) External view of the right posterior segment showing a well-circumscribed mass. (B) Cut surface of the bisected specimen demonstrating a lobulated lesion with heterogeneous, tan-white appearance and focal areas of cystic change.

Histologic examination revealed an infiltrative high-grade neoplasm composed of sheets of large, discohesive cells in a vascular stroma. Tumor cells exhibited abundant eosinophilic, sometimes vacuolated cytoplasm; oval to irregular nuclei with vesicular chromatin; prominent nucleoli; marked pleomorphism; and numerous mitoses. Extensive necrosis, scattered lymphocytes, and delicate fibrous septa were present. The infiltrative borders and sinusoidal growth pattern resembled previously described hepatic histiocytic proliferations. All surgical margins were free of tumor (Figure 3).

**Figure 3.**
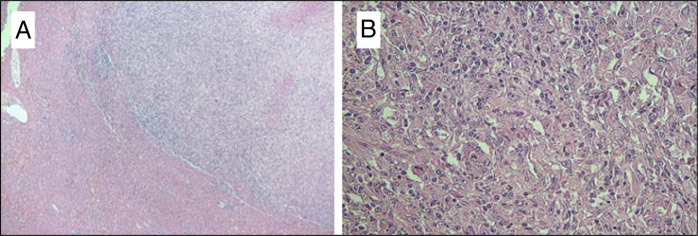
Microscopic views of the hepatic lesion. (A) Low-power view (4×) showing an infiltrative neoplasm permeating the hepatic parenchyma. (B) Higher-magnification view (40×) highlighting the discohesive large cells and high-grade cytological features characteristic of the tumor.

Immunohistochemical analysis showed strong and diffuse positivity for the histiocytic markers CD68 and CD163, with additional positivity for CD45, supporting a hematopoietic origin. By contrast, the lesion was negative for epithelial markers (cytokeratin AE1/AE3, EMA, HepPar-1), vascular markers (CD34, ETS‑related gene [ERG], human herpesvirus‑8), and markers associated with specific sarcoma subtypes (MDM2, CDK4, h-Caldesmon, MyoD1, ALK). Lymphoid markers (CD20, CD30) were also negative. S-100 expression was limited to scattered dendritic-appearing cells, consistent with background staining rather than true tumor positivity. The proliferation index was markedly elevated, as demonstrated by a high Ki-67 labeling fraction.

The strong expression of histiocytic markers CD163 and CD68, along with the absence of markers of Langerhans cells (CD1a, CD207), follicular dendritic cells (CD21, CD35), myeloid blasts (MPO, CD34), melanocytic markers, and lineage-defining B-cell or T-cell antigens, meets the essential immunophenotypic criteria for HS as outlined in the WHO classification. Although occasional S-100–positive dendritic-appearing cells were present, there were no morphologic features to support a Langerhans cell or follicular dendritic cell lineage, such as nuclear grooves typical of Langerhans cells or the fascicular/spindled architecture of dendritic cell tumors (Figure 4).

**Figure 4.**
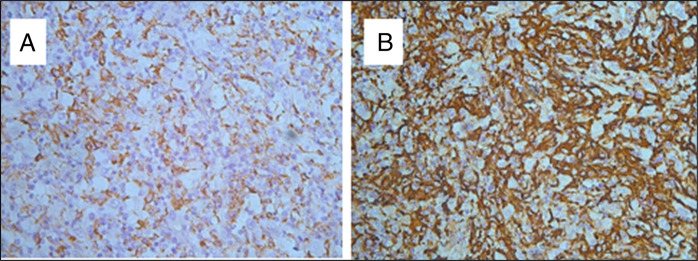
Immunohistochemical staining for CD68. (A) Low-power view (4×) showing diffuse CD68 positivity within the infiltrative neoplasm. (B) High-power view (40×) highlighting strong cytoplasmic expression in the neoplastic cells.

The patient's postoperative course was initially uneventful. The abdominal drain was removed on postoperative day 3, and he was discharged home in stable condition on postoperative day 5. However, over the following weeks, he experienced rapid clinical deterioration. Imaging during the first 2 postoperative months revealed extensive disease progression, precluding the initiation of systemic therapy. Despite supportive care, the patient's condition continued to decline, and he died approximately 3 months after surgery.

## DISCUSSION

HS is an exceptionally rare hematopoietic malignancy defined by morphologic and immunophenotypic features of mature histiocytes. It affects a broad age range, with a median age of approximately 52 years and a slight male predominance. HS may arise in lymph nodes, skin, soft tissues, gastrointestinal tract, liver, and spleen According to the WHO classification, it is characterized by the morphologic and immunophenotypic profile of mature tissue histiocytes.^1,3^

Diagnosis requires comprehensive pathological evaluation within the appropriate clinical context. Despite advances in phenotypic and molecular characterization, HS remains diagnostically challenging, and accurate classification depends on confirming histiocytic lineage while excluding other poorly differentiated large-cell malignancies.^3–6^

Recent studies highlight the importance of molecular alterations in HS pathogenesis. Recurrent mutations in the MAPK/ERK pathway,^7^ including *BRAF, MAP2K1*, and *KRAS*, promote proliferation and survival of neoplastic histiocytes.^8,9^ Additional abnormalities involving the PI3K/AKT pathway^8,^ and tumor suppressor genes such as *TP53*^10^ have been described. These findings support a model of dysregulated cell-cycle control and possible transdifferentiation from hematopoietic progenitors or evolution from antecedent hematologic malignancies.^11^ MAPK pathway mutations likely contribute to the rapid growth and high-grade cytology characteristic of HS.^12^ As molecular profiling becomes more accessible, it may refine diagnosis and identify potential therapeutic targets.

Clinically, HS presents with nonspecific symptoms and significant morphologic overlap with other large-cell malignancies, contributing to diagnostic uncertainty. Approximately half of cases arise in soft tissues, while one-third involve the gastrointestinal tract; secondary hepatic or nodal involvement is also common.^13,14^

From an imaging perspective, several hepatic lesions may mimic the appearance of the mass identified in segment VI. Lesions demonstrating peripheral or irregular enhancement on MRI often raise consideration of intrahepatic cholangiocarcinoma, which typically presents with lobulated margins, delayed progressive enhancement, and associated capsular retraction.^15^ Atypical HCC may also enter the differential, particularly when arising in noncirrhotic livers, although the absence of nonrim arterial phase hyperenhancement and nonperipheral washout makes classic HCC less likely.^16^ Metastatic disease—especially from primaries such as melanoma, sarcoma, or gastrointestinal tumors—can present as T2-hyperintense lesions with restricted diffusion and peripheral enhancement, and should be considered in patients with compatible clinical backgrounds.^17^ Other mimickers include hepatic abscesses, which may show T2 hyperintensity and peripheral enhancement but are usually accompanied by systemic signs of infection, and hemangiomas, although their characteristic centripetal fill-in pattern was not observed here.^18^ Given the lesion's exophytic growth pattern and location in segment VI, adrenal rest tumors^19^ and solitary fibrous tumors^20^ may also be considered, although these entities are rare. Overall, the constellation of imaging findings in this case favored a malignant process but lacked the hallmark features of HCC, necessitating a broad imaging-based differential diagnosis.

Histologically, HS consists of large pleomorphic cells with eosinophilic cytoplasm and prominent nucleoli. Immunohistochemically, neoplastic cells express macrophage-associated markers such as CD68, CD163, or lysozyme, while lacking markers of lymphoid, dendritic, epithelial, muscle, and myeloid lineages—an essential feature for excluding mimickers including dendritic cell sarcoma, diffuse large B-cell lymphoma, anaplastic large T-cell lymphoma, myeloid sarcoma, undifferentiated carcinoma, and melanoma.^13,21–23^

For localized disease, complete surgical resection is the preferred treatment. However, HS often follows an aggressive course. No standardized systemic therapy exists; Cyclophosphamide, doxorubicin, vincristine, and prednisone–based regimens are most commonly used,^24^ although responses are variable. More intensive protocols such as ifosfamide, carboplatin, and etoposide, etoposide, prednisone, vincristine, cyclophosphamide, and doxorubicin, or acute myeloid leukemia–type regimens have been attempted,^25^ and isolated reports describe responses to cladribine or immune checkpoint inhibitors. Overall prognosis remains poor, with median survival ranging from months to 2 years depending on disease burden and treatment response.^26,27^

In summary, this case adds meaningful insight to the very limited literature on hepatic HS, an exceptionally rare malignancy with highly variable presentation. Our report is particularly notable for its unique manifestation as an isolated hepatic lesion, a pattern seldom described in prior publications. The case also underscores the absence of disease-specific imaging criteria, as the lesion demonstrated nonspecific MRI features and an LR-M pattern on LI-RADS assessment, making preoperative diagnosis challenging. Most importantly, the extraordinarily rapid disease progression, despite complete (R0) surgical resection, highlights the aggressive nature of this entity. The patient's fulminant clinical decline and death within 3 months of surgery emphasize the need for ultra-rapid referral pathways and expedited multidisciplinary management once hepatic HS is suspected. Collectively, these observations reinforce the importance of reporting individual cases to improve recognition, diagnostic accuracy, and timely intervention for this rare and aggressive disease.

## DISCLOSURES

Author contributions: E. Tziava collected the clinical information, performed the literature review, and drafted the manuscript. A. Leventi contributed to manuscript writing, revised the text, and prepared the images. M. Arnaouti and N. Ntalakos contributed to manuscript writing and prepared the pathology images. M. Igoumenidi assisted with data collection and contributed to the literature review. DP Korkolis conceived the study, contributed to data collection, and assisted in drafting and revising the manuscript. DP Korkolis is the article guarantor.

Financial disclosure: None to report.

Informed consent was obtained for this case report.

## ABBREVIATIONS:

AFP: alpha‑fetoprotein
ALK: anaplastic lymphoma kinase
AML: acute myeloid leukemia
CA19‑9: carbohydrate antigen 19‑9
CD: cluster of differentiation
CEA: carcinoembryonic antigen
CHOP: cyclophosphamide, doxorubicin, vincristine, prednisone
CT: computed tomography
EMA: epithelial membrane antigen
EPOCH: etoposide, prednisone, vincristine, cyclophosphamide, doxorubicin
ERG: ETS‑related gene
HCC: hepatocellular carcinoma
HHV‑8: Human herpesvirus 8
HS: histiocytic sarcoma
ICE: ifosfamide, carboplatin, etoposide
KI‑67: proliferation index marker Ki‑67
KRAS: Kirsten rat sarcoma viral oncogene homolog
LI‑RADS: liver imaging reporting and data system
LR‑M: LI‑RADS category: probably or definitely malignant, not specific for HCC
MAP2K1: mitogen‑activated protein kinase kinase 1
MAPK: mitogen‑activated protein kinase
MDM2: mouse double minute 2 homolog
MPO: myeloperoxidase
MRI: magnetic resonance imaging
PI3K/AKT: phosphatidylinositol‑3‑kinase / protein kinase B pathway
S‑100: S‑100 protein family
TP53: tumor protein p53
WHO: World Health Organization
